# Isolation and genetic characterization of metallo-β-lactamase and carbapenamase producing strains of *Acinetobacter baumannii* from patients at Tehran hospitals

**Published:** 2011-06

**Authors:** F Shahcheraghi, M Abbasalipour, MM Feizabadi, GH Ebrahimipour, N Akbari

**Affiliations:** 1 Department of Bacteriology, Pasteur Institute of Iran, Tehran, Iran; 2 Faculty of Biology Science, Shahid Beheshti University, Tehran, Iran; 3 Department of Microbiology, School of Medicine, Tehran University of Medical Sciences, Tehran, Iran

**Keywords:** *Acinetobacter*, betalactamase, metallo beta lactamase, OXA-type

## Abstract

**Background and Objective:**

Carbapenems are therapeutic choice against infections caused by gram-negative bacilli including strains of *Acinetobacter baumannii*. Resistance to these antibiotics is mediated by efflux pumps, porins, PBPs and ß-lactamases. The aim of this study was to determine the possibility of existence of MBLs, OXAs and GES-1 betalactamase genes among clinical isolates of *Acinetobacter* collected from Tehran hospitals.

**Material and Methods:**

Two hundred and three *Acinetobacter* isolates were collected from patient at Tehran hospitals. The isolates were identified using biochemical tests. The susceptibility to different antibiotics was evaluated by disk diffusion method and MICs of imipenem were determined using Micro broth dilution method (CLSI). PCR was performed for detection of *bla*_VIM-2_, *bla*_SPM-1_, *bla*_IMP-2_, *bla*_GES-1_, *bla*_OXA-51_, *bla*_OXA-23_ betalactamase genes. Clonal relatedness was estimated by PFGE with the restriction enzyme *Sma*I.

**Results and Conclusion:**

Of 100 isolates of imipenem resistant *Acinetobacter* spp. collected from Tehran hospitals in 2009 and 2010, 6 isolates produced metallo-beta-lactamase**s** and 94 isolates produced OXA-type carbapenemase. The *bla*_SPM-1_, *bla*_GES-1_, *bla*_OXA-51_, *bla*_OXA-23_ genes were detected by PCR among 6, 2, 94 and 84 isolates of *A. baumannii*, respectively. The MICs of isolates to imipenem were 8–128 µg/mL. PFGE analysis of 29 *bla*_OXA-51_ and *bla*_OXA-23_-positive *A. baumannii* isolates gave 6 different patterns. This is the first report of SPM-1 and GES-1 beta-lactamase producing *A. baumannii*. Production of the OXA-23, OXA-51, GES-1 and SPM-1 enzyme presents an emerging threat of carbapenem resistance among *A. baumannii* in Iran.

## INTRODUCTION

*Acinetobacter* is an important cause of nosocomial infections and has been associated with a wide variety of illnesses in hospitalized patients, especially patients in the intensive care units (1). The clinical strains of *A. baumannii* are usually multidrug resistant to aminoglycosides, fluoroquinolones, ureidopenicillins and third generation cephalosporins. In cases of resistance to betalactams caused by extended spectrum β-lactamases and AmpC enzyme, carbapenems are often used as the last resort against infections. However, carbapenem-hydrolyzing-β-lactamases of molecular class B and D have emerged over the last decade (2).

Class B carbapenemases including IMP and VIM termed as metallo beta lactamases (MBLs) have been found so far in *A. baumannii* and these are encoded by different plasmid types (3). Class D (OXA) have emerged as the major carbapenemases in the world.OXA enzyme (encoded by *bla*_OXA_ genes) can be sub-classified into eight subgroups, of which OXA-23-like, OXA-24-like, OXA-51-like and OXA-58-like have been identified in *A. baumannii*. The *bla*_OXA-51_^-^type genes are intrinsically harbored by *A. baumannii* isolates (4). Recently, new classes of betalactams including SPM-1, GIM-1, SIM, and AIM have also been reported. GES-2, GES-4 and GES-5 class A β-lactamases showed weak activity for hydrolysis of imipenem (5).

The first nosocomial outbreak with carbapenem-resistant *A. baumannii*) CRAB)was reported from the USA in 1991. Since then, CRAB infections and extensive hospital outbreaks have been reported throughout the world (6). However, despite the worldwide occurrence of epidemic carbapenem resistance strains, metallo beta lactamase producing *A. baumannii* isolates have been found to be disseminated only in specific geographic areas (7). Gram negative bacilli producing acquired IMP, VIM and SPM-1 has been reported more in Asia and South America, respectively (8). Because of the importance of resistance to beta lactam antibiotics among clinical isolates, understanding the underlying genetic mechanisms responsible for the acquisition and spread of unique beta lactamase-mediated antibiotic resistance mechanism could eventually facilitate the development of effective prevention and control measures (9).

Recent studies have shown the existence of different MBLs producing clones of *A. baumannii* in Iran. The aims of this study were to determine the drug susceptibility patterns of *Acinetobacter* spp. isolated from patients at different Tehran hospitals and to identify the genes encoding VIM, IMP, SPM and GES carbapenemase among the isolates. Pulsed-Field Gel Electrophoresis (PFGE) was then used to investigate the genetic relationships among the isolates and possibility of intra-and inter-hospital spread of resistant strains.

## MATERIALS AND METHODS

**Bacterial identification and susceptibility testing.** Two hundred and three non-repetitive isolates of *Acinetobacter* recovered from blood, wound, urine, sputum, and respiratory tract were obtained from 7 hospitals in Tehran between May 2009 to September 2010. The isolates were identified by conventional biochemical methods and confirmed by PCR assay targeting the *bla*_OXA-51_ like gene (10).

All imipenem-resistant isolates were also tested in the Kirby Bauer method of disk diffusion to check their susceptibilities to aztreonam (ATM: 30 µg), amikacin (AN: 30 µg), cefepime (CPM: 30 µg) cefotaxime (CTX: 30 µg), ceftazidime (CAZ: 30 µg), ceftriaxone (CRO: 30 µg), cefexime (CFM: 30 µg), ciprofloxacin (CIP: 5 µg), meropenem (MEM: 10 µg), imipenem (IMP: 10 µg), piperacillin (PRL: 100 µg), piperacillin-tazobactam (PTZ: 110 µg), colistin (CL: 10 µg), polymixinB (PB: 300 unit) (MAST, Merseyside, U.K). Multidrug resistance (MDR) was defined as resistance to 3 or more classes of drugs that would otherwise serve as treatments for *Acinetobacter* infection (e.g., quinolones, cephalospo-rins, and carbapenems) (11, 12).

Strains found resistant to imipenem by disk diffusion test were re-checked in broth micro dilution assay (Sinha *et al*., 2007). MIC for imipenem ranging from 0.25 µg/ml through 128 µg/ml was tested. ATCC *Pseudomonas aeruginosa* 27853 was used as control strain. The MICs≥8 µg/ml was interpreted as resistance to imipenem (13).

**PCR assay**. DNA template from imipenem resistant isolates (MIC≥8 µg/ml) were extracted by boiling for 15 minutes and used as template in PCR assay to amplify *bla*_VIM-2_, *bla*_SPM-1_, *bla*_IMP-2_, *bla*_GES-1_, *bla*_OXA-51_, *bla*_OXA-23_ genes by using thermocycler (Eppendorf, Hamburg, Germany). The primers described by Shibata, Weldhagen and Woodford were used in this experiments (14, 15, 4). The reactions were initiated in solution containing 200 µM concentrations of dNTPs, 10 pM of each primer, 0.8 mM MgCl_2_, 0.5U Taq polymerase (Metabion, Martinsried, Germany) and 50 ng DNA template in a final volume of 25 µL.

*A. baumannii* AC54/97 producing *bla*_IMP_ gene (16), *P. aeruginosa* PO510 producing *bla*_VIM-1_, *P. aeruginosa* COL-1 producing *bla*_VIM-2_, *P. aeruginosa* 16 producing *bla*_SPM-1_ (Kindly provided by P. Nordmann), *K. pneumoniae* ORI-1 producing *bla*_GES-1_, *A. baumannii* NCTC 13304 and A. baumannii NCTC # 12156 producing *bla*_OXA-23_ and *bla*_OXA-51_ were used as controls.

The amplicons were electrophoresed in 1% agarose gel and visualized after staining with ethidium bromide. A 100 bp ladder (Fermentas, Vilnius, Lithuania) was used as molecular weight marker. The PCR products for *bla*_GES-1_, *bla*_SPM-1_, *bla*_OXA-51_ and *bla*_OXA-23_ were purified on Qiaquick columns (QIAGEN, Ca, USA) and the sequencing was carried out using the ABI capillary system (Macrogen Research, Seoul, Korea).

The nucleotide sequences of amplicons have been assigned to the Gene Bank under accession numbers HM370522, HM370523, HQ222987, HQ222988 for *bla*_GES-1_, *bla*_SPM-1_, *bla*_OXA-51_ and *bla*_OXA-23_ respectively.

**Plasmid DNA extraction and conjugation experiments. **Plasmid DNAs from the *blaSPM* and *blaGES* positive strains of *A. baumannii* were extracted using the plasmid extraction kit (Qiagen, Courtaboeuf, France) and targeted for the *blaSPM* and *blaGES-1* genes using the primers described previously. Transfer of resistance by conjugation was attempted using *A. baumannii* (donor) and *E. coli* K12 (recipients). Overnight filter mating experiments were performed at 37°C, and the transconjugants were selected on MacConkey agar and plates supplemented with imipenem (16 µg/ml).

**MBL screening.** Screening for MBLs in selected isolates was performed with combined disk synergy test. In brief, disks containing 750 µg of EDTA plus 10 µg of imipenem were placed on the inoculated plates containing Muller Hinton agar. An increase of≥17 mm in zone diameter in the presence of 750 µg of EDTA compared to imipenem alone indicated the presence of an MBL (9).

**Fig. 1:**
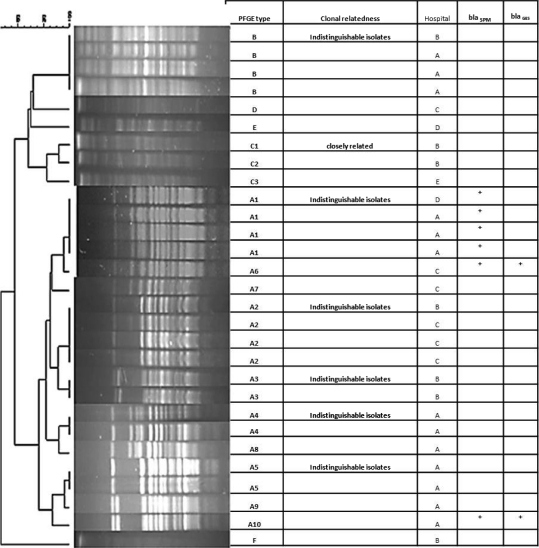
Dendrogram of the six (A–F) pulsed field gel electrophoresis profile of Smal-digested genomic DNAfrom 26 blaOXA-23 positive A. baumannii isolates were from hospital A to E.

**PFGE.** The intact chromosomal DNAs from 29 MDR imipenem-resistant *Acinetobacter baumannii* carrying *bla*OXA-23 (MICIMP≥8 µg/ml) belonging to the same antibiotype were extracted for PFGE as describ-ed by Durmaz et al**. (17). These strains collected from 5 hospitals where the most collected isolate came from. The PUlseNet universal standard marker strain *Salmonella choleraesuis* serotype *Branderup* H9812 was used as a molecular size marker. The gels were stained with ethidium bromi-de and DNA patterns were photographed with UVP gel Documentation (UVP, UK) (Fig. 1). The DNA banding patterns were analyzed using GelCompare II software (Apllied maths NV, St-Martens-Latem Beligum). PFGE DNA pattern was compared and interpreted according to criteria of Tenover et al**. (18).

**Statistical Analysis.** Description and analysis of the data were carried out using SPSS 16.0 software. The nonparametric Mann-Whitney test was used for comparison between the different groups. A p-value≤0.05 was considered significant.

## RESULTS

**Clinical bacterial strains. **Of 203 isolates of *Acinetobacter* spp. cultured from clinical specimens, 100 were resistant to imipenem by disk diffusion method and these were cultured from blood (47%), respiratory tract (24%), wound (12%), sputum (10%) and urine (7%). The results of broth microdilution assay showed high-level of resistance to imipenem. Most of the impenem-resistant isolates (*n=*100) were also resistant to aztreonam (96%), amikacin (84%), cefepime (90%), cefotaxime (98%), ceftazidime (86%), ceftriaxone (99%), cefexime (100%), ciprofloxacin (84%), meropenem (100%), piperacillin (96%), piperacillin-tazobactam (95%), colistin (12%) and polymixinB (3%). Totally 83 isolates were recognized as MDR (Multi Drug Resistance). Taking the MICs of≥8 µg/mL as resistant, all studied isolates were resistant to imipenem. An MIC=64 µg/mL was found among 47% of MDR isolates.

A total of 100 selected isolates were examined for MBL profiles by the combined-disk method. This method showed that 9% of isolates were able to produce MBLs, of which 6 strains were identified as *A. baumannii* and 3 isolates belonged to the other species of *Acinetobacter*.

Using PCR assay, 94% and 84% of the isolates were positive for *bla*OXA-51 and *bla*OXA-23 like genes respectively. Of 100 isolates tested, 6 strains (ABI/30, ABI/41, ABI/43, ABI/51, ABI/58 and ABI/70) and 2 strains (ABI/43 and ABI/51) appeared to carry *bla*SPM-1 and *bla*GES-1 respectively (Table 1). The *bla*VIM-2 and *bla*IMP genes were not detected among the studied isolates.

**Table 1:** Characterization of *bla*_SPM-1_ and *bla*_GES-1_ positive *Acinetobacter* clinical isolates.

Name of Hospital	Antibiotic resistance												MICIMPg/ml				PCR	I	Isolates

		ATM	CRO	CPM	AN	CAZ	CIP	CTX	PRL	MEM	IMP	CFM	CPTZ	CL	PB		OXA-51,23	SPM	GES
**ABI/30**	D	R	R	R	S	R	R	R	R	R	R	R	R	S	S	≥128	+	+	−

**ABI/41**	A	R	R	R	S	R	R	R	R	R	R	R	R	S	S	64	+	+	−

**ABI/43**	C	R	R	R	S	R	R	R	R	R	R	R	R	S	S	64	+	+	+

**ABI/51**	A	R	R	R	S	R	R	R	R	R	R	R	R	S	S	64	+	+	+

**ABI/58**	A	R	R	R	S	R	R	R	R	R	R	R	R	S	S	64	+	+	−

**ABI/70**	A	R	R	R	S	R	R	R	R	R	R	R	R	S	S	64	+	+	−

“Abbreviations: ATM=Aztereonam, CRO=Ceftriaxone, CPM=Cefepime, AN=Amikacin, CAZ=Ceftazidime, CIP=Ciprofloxacin, CTX=Cefotaxime, PRL=Pipercillin, MEM=Meropenem, IMP=Imipenem, CFM=Cefexime, PTZ=Pipercillin- tazobactam, Cl=Colistin, PB=Plolymixin B

All of the *bla*SPM positive isolates were collected from different hospitals and their antibiogram indicat-ed that our studied isolates were resistant to all studi-ed antibiotic except polymixin B and colistin. Only two isolates (ABI/43 and ABI/51) carried *bla*SPM, *bla*GES, *bla*OXA-51and *bla*OXA-23 simultaneously (Table 1).

**Plasmid DNA extraction, conjugation experi-ments.** PCR results show that *blaS*PM-1 and *bla* GES-1 are carried by plasmid. Our experiments for conjugal transformation of plasmid did not give any result.

**Sequencing. **Aligning of the obtained sequences with those of reference strains in Gene Bank confirm-ed the correct identification of *bla*GES-1, *bla*SPM-1, *bla*OXA-51 and *bla*OXA-23 by PCR.

**PFGE analysis. **Totally, 29 isolates with MDR phenotype were analyzed by PFGE and these were collected from hospitals A (n=13), B (n=7), C (n=6), D (n=2) and E (n=1). They showed co-resistance to antimicrobial agents including amikacin, aztreonam, piperacillin, piperacillin–tazobactam, ceftazidime, cefepime, cefexime, cefotaxime, cefteriaxone, ciprofloxacin, meropenem, polymyxin B and colistin. Genotyping identified 6 distinct pulsotypes allocated as A to F among 5 hospitals (Fig. 1). The pulsotypes A, B and C were the dominant types found in the hospitals. Conversely, pulsotypes D, E and F each contained single isolate. The pulsotype A consisted of 19 isolates. They were differentiated into closely related pattern comprising of patterns A1–A10 (Fig. 1). Isolates from hospital A were heterogeneous since 7 different banding patterns were found for 13 isolates. More diversity were found among the isolates at hospitals B (n=7) and C (n=6) since 6 and 4 pulsotypes were found among the isolates of these hospitals respectively (Fig. 1). Six SPM-1-producing and two GES-1-producing isolates produced closely related patterns.

## DISCUSSION

This is the first report on the existence of *bla*SPM-1 and *bla*GES-1 among the clinical strains of*A. baumannii* in Iran. Occurrence of SPM-1 MBL among *P. aeruginosa* has been described among clinical isolates in Brazil. SPM-1 is distinct from VIM and IMP and represents a new subfamily of mobile MBL which is carried on a plasmid. This is important since it can transform both *E. coli* and *P. aeruginosa* to ceftazidime resistance (19). Detection of GES-1 among the Iranian strains of *A. baumannii* is another important finding of the current study. This ESBL enzyme was initially described from a *Klebsiella pneumoniae* strain in 1998. GES-1 is weakly related to the other Ambler class A β-lactamases, particularly to the plasmid-located ESBLs so far identified in *Enterobacteriacae*. However, GES-1 may be classified rather as a ceftazidime-hydrolyzing enzyme (20). Our survey showed that these two genes can not be transferred to *E. coli* K12 strain by conjugation.

While IMP-2 is reported in *A. baumannii* from Italy and Japan, VIM enzymes have been identified very rarely in *A. baumannii*, being represented only by VIM-2 reported in South Korea. Despite carbapenemase activity of resistant strains, these MBL genes have not been detected in *A. baumannii* from Iran (21). Among MBL genes, only VIM-type had been detected in *P*. *aeruginosa* in Iran (16).

The MBLs can hydrolyze all beta-lactams except aztreonam. However, all MBL producing isolates in our study were resistant to aztreonam too. Other mechanisms such as ESBL production, efflux pumps and hyper-production of cephalosporinase possibly are involved in resistance to aztreonam in our collection of isolates.

This study showed low susceptibility rates to most of the clinically available antimicrobial agents for the treatment of infections caused by *Acinetobacter* spp. except for polymyxin B and colistin. This finding suggests both of them as therapeutic options for the treatment of infections caused by these isolates. Among imipenem resistant isolates, 94% harboured at least one acquired class D carbapenemase-encoding gene. Therefore, the high prevalence of class D carbapenemase-encoding genes among the clinical isolates from Iran appears responsible for low susceptibility rates for imipenem [*P<*0.05; sig=0.002]. According to the SENTRY report distribution of OXA-type genes among *Acinetobacter* spp. isolates in Asia-Pacific nations was comprised mainly of *bla*OXA-23, while *bla*OXA-24/40 and *bla*OXA-58 were less common. In addition, clonal dissemination was found among different medical centers in different countries in the Asia-Pacific region (22). In our study, *bla*OXA-23 was the most common genes encoding carbapenamase at Tehran hospitals.

PFGE analysis revealed genetic diversity among the MDR *A. baumannii* isolates. These results may support the assumption that MDR strains are emerged more by horizontal transfer of MBL genes among different phenotypic clones of *Acinetobacter*. Six SPM-1-producing and two GES-1-producing isolates showed closely related patterns. Such a correspondence of phenotypic and genotypic characteristic can be attributed to a common clonal origin. Some isolates that gave identical typing results had been isolated from patients of different hospitals at different times. Therefore, these types have simultaneous presence and genetically are stable in community. Moreover, a few of these isolates showed different antimicrobial susceptibility patterns. This caused by transfer of these antibiotic resistance genes to the similar clonal strains through transformation or conjugation. The possibility of inter-hospital transmission of potentially pathogenic bacteria including *P. aeruginosa* has already been debated in Iran (23).

In conclusion, the rate of resistance to betalactams was high in our study. Since the rate of isolates carrying *bla*SPM-1and *bla*GES-1 was very low, it seems that other mechanisms such as decreased permeability, over expression of efflux pump, production of carbapenemases probably are involved in resistance to betalactams. Understanding the underlying genetic mechanisms responsible for the acquisition and spread of this unique beta lactamase mediated antibiotic resistance mechanism could eventually facilitate the development of effective prevention and control strategies and thereby allowing more effective drug usage and treatment of disease, and reducing resistance development (9).
